# Gamma rays and sodium azide induced variations in bio-physiological and agronomical traits in linseed (*Linum usitatissimum* L.)

**DOI:** 10.1016/j.heliyon.2024.e31329

**Published:** 2024-05-16

**Authors:** Roshan Jahan, Aamir Raina, Saima Malik, Samiullah Khan

**Affiliations:** a Mutation Breeding Laboratory, Department of Botany, Aligarh Muslim University, Aligarh, 202002, Uttar Pradesh, India; b Botany Section, Women's College, Aligarh Muslim University, Aligarh, 202002, Uttar Pradesh, India

**Keywords:** Linseed, Mutagenesis, Gamma rays, Sodium azide, Bio-physiological parameters

## Abstract

Linseed is a valuable oilseed crop with huge therapeutic importance due to its high content of omega-3 fatty acids in the form of Alpha-linolenic acid (ALA). It is a self-pollinated crop with a low-yielding potential that restricts its improvement endeavors. To overcome low-yielding potential, individual and combination treatments of gamma rays and sodium azide were employed in widely grown linseed varieties. The results revealed a dose-dependent decline in seed germination, seedling height, pollen fertility, chlorophyll, and carotenoid contents and a dose-independent decline in carbonic anhydrase activity. Bio-physiological parameters decreased substantially in combination treatments compared to individual treatments of gamma rays and sodium azide. In contrast, lower doses of gamma rays, sodium azide, and their combinations effectively increased mean values of yield and yield-attributing traits in a few putative mutants. Such putative mutants represent a valuable genetic resource that could be used in future breeding programs for the genetic improvement of linseed and related medicinal plants.

## Highlights

• The high-yielding mutant lines of linseed developed in the present study could serve as valuable genetic resources in crop improvement programs.
• Lower doses of gamma rays and sodium azide effectively increased the mean values of important traits.
• Mutagens increased the genotypic coefficient of variability, heritability, and genetic advance in high-yielding mutant lines.

## 1 Introduction

Linseed is a self-pollinated, annual diploid (2n = 2x = 30) herbaceous oilseed crop with a narrow genetic base [1]. It belongs to the genus *Linum* and the family Linaceae, which comprises 300 species [2]. The estimated genome size of linseed is approximately ∼380 Mbp [3]. It is a winter crop that requires moderate to cool temperatures during the growing season and can be raised in various soils where enough moisture is available. Moderate rainfalls are suitable for cultivating linseed, and high temperatures during the flowering stage may reduce the seed yield and oil content [4]. Linseed is planted from October to November, depending on the soil moisture availability, and harvested from April to May. Linseed is nutritionally rich and contains protein content (18.00 g), fibre (23.1 g), total lipid (42.16 g), carbohydrates (34.4 g), and essential mineral elements like potassium (793 mg), phosphorus (556 mg), calcium (230 mg), magnesium (372 mg), sodium (37 mg), iron (5.78 mg) per 100 g (**Source:** USDA; National Nutrient Database for Standard Reference, 2023). Linseed is gaining popularity due to its high content of omega-3- fatty acids (Alpha-linolenic acid), which are essential for human health. The main bioactive components in linseed include 55 % of Alpha-linolenic acid (ALA), 30 % of protein, and 35 % dietary fibre [5]. Linseed has proven its various beneficial roles, including preventing cardiovascular disease [6,7], inflammatory disorders [8], rheumatoid arthritis [9], diabetes [10], autoimmune disorders [11], hormonal imbalance [12], and several types of cancer [[13], [14], [15], [16]]. Growing interest indicates that linseed remains a multi-purpose crop globally grown for various benefits in several countries, including the United States of America, Russia, India, China, Canada, and Ethiopia. Annually, linseed is cultivated on 3.79 million hectares (mha), producing 3.69 million tonnes (mt) (https://www.fao.org, accessed on November 02, 2022). According to the Food and Agriculture Organization Statistics Division 2023, Asia has the highest linseed producers, contributing up to 40.6 % of total linseed production worldwide. The top producers of linseed (average data of 2010–2020) include Kazakhstan (0.54 mt), Canada (0.59 mt), Russian Federation (0.49 mt), China (0.36 mt), China mainland (0.36 mt), United States of America (0.15 mt), India (0.14 mt) and Ethiopia (0.09 mt). India was the 7^th^ most producer of linseed after the USA; however, the yield of linseed in India (5046 hg/ha (hg/ha) is low compared to the world average (9703 hg/ha), which indicates a narrow genetic base of available linseed genotypes and the scarcity of effective breeding strategies. Conventional breeding methods have been successful in increasing linseed yield; however, it requires more time and labour. As a result, increasing genetic variability in less time appears daunting [17,18]. Modern breeding techniques should be implemented at a large scale to overcome the bottleneck of conventional breeding methods. Among modern breeding approaches, mutation breeding has been a cost-effective, coherent, and sophisticated technique to enhance accessible genetic variability for developing mutants with desirable agro-economic traits [19,20]. Mutation breeding has emerged as a successful strategy for developing thousands of improved cultivars [21]. However, mutation breeding has drawbacks, such as mutations being random in nature, making an entire breeding approach a hit-and-trial method. In addition, mutations are induced at a low frequency that requires a sharp breeder's eye to spot a single mutant in thousands of wild plants. Despite limitations, mutation breeding has been effective in enhancing agro-economic traits in various crops, such as *Nigella sativa* [22], *Lens culinaris* [23], and *Vigna mungo* [24]. However, only eleven linseed mutant varieties have been developed, indicating that linseed has received less attention from scientists (https://mvd.iaea.org accessed on March 24, 2024). This may be due to the dearth of literature on optimizing doses of gamma (γ) rays, sodium azide (SA), and γ rays + SA that could be employed for the linseed improvement. A variable range of γ rays has been reported to play a role in the linseed improvement that leads to much ambiguity in selecting the optimum dose of γ rays. In the case of chemical and combined mutagen doses, not a single individual or combined dose of gamma rays and sodium azide has been reported successful in developing an elite mutant variety of linseed. In 2019, high-yielding and early flowering linseed mutant lines number 85 and 66 were developed in India by treating seeds with 0.3 % EMS. In 1965, a high oil content mutant variety, viz., Redwood 65, was developed in Canada by irradiating seeds with x-rays. In 1979, a high oil content linseed mutant variety viz., Dufferin was developed in Canada. In 1978, the linseed mutant variety, Heiya 4, showed early maturity and tolerance against lodging, moisture, salinity, and alkalinity. In 1985, Heiya 6 was also developed in China by the hybridization of two mutants obtained by irradiation with 200 and 300 Gy gamma rays. The improved attributes of this mutant variety are high yield, resistance to lodging, diseases, salinity, and alkalinity. In 1989, a high-yield and good-quality mutant variety, Heiya 7, was developed in China by irradiation with gamma rays (100 Gy). In 1996, a high oil-quality mutant variety Linola 989 was developed in China by hybridization with the Australian mutant variety Zero low linolenic acid. In 1982, early maturity, lodging resistant, and good quality mutant variety viz., Ningya 10 was developed in China by irradiating seeds with gamma rays (100Gy). In 1991, lodging, fusariosis, anthracnose, and bacteriosis-resistant linseed mutant variety viz., M−5 was developed in the Russian Federation by treatment of seeds with a water solution of DMS (0.05 %). In 1988, a late maturity, good quality, and high yield mutant variety, viz., Zarja 87, was developed in the Russian Federation by treating hybrid seeds (LD-147 x Complex) with a water solution of ethylene imine. In 1991, another lodging and fusariosis-resistant linseed mutant variety, Baltyuchai, was developed in the Russian Federation by treating seeds with N-nitrosoethyl urea (0.012 %). After reviewing the literature, we realized that there was a dearth of literature on optimum doses of gamma rays and sodium azide that could be used to improve the yield and oil content of existing linseed varieties. Therefore, we designed and implemented mutagenesis followed by extensive phenotypic selection over multiple years to increase genetic variation in the linseed genome. Genetic variation is a prerequisite for any crop improvement program, genetic variation induced by mutagens could also be pivotal in improved varieties [25]. Mutagens are suitable agents for increasing genetic variation in crops. Among the mutagens, gamma rays are widely used due to their ability to interact with plant tissues and generate highly reactive radicals that eventually interact with nucleic acids [[26], [27], [28]]. Besides gamma rays, chemical mutagens also became popular due to their ease of use and less dependence on sophisticated laboratory instruments [[29], [30], [31], [32]]. Among chemical mutagens, sodium azide (NaN_3_) is capable of inducing a point mutation and altering genetic information [31,33]. Several workers have employed sodium azide in improving the agronomy of various crops, such as maize [34], cowpea [35], linseed [36], wheat [37], and fenugreek [38]. Single and combined mutagen treatments have been used to enhance the agro-economic traits in various plants, including oilseed crops, pulses, cereals, and ornamental plants. Different physical and chemical mutagens used alone or in combinations can modify DNA bases, break DNA strands, and distort the double helical structure [39]. Considering the remarkable results of gamma rays, sodium azide, and their combinations in improving the crops, we treated two linseed varieties with different doses of gamma rays and sodium azide.

## 2 Materials and methods

### 2.1 Experimental materials

Linseed varieties Padmini and IC0096650 were procured from the Governmental Seed Store, Aligarh, and the National Bureau of Plant Genetic Resources (NBPGR), New Delhi, respectively (Supplementary Table 1).We found that the agro-climatic conditions of North India, including Aligarh, are suitable for both varieties after raising several generations.

### 2.2 Mutagen treatment

In both the varieties of linseed (Padmini and IC0096650) eight sets comprising 300 seeds each were treated with different doses of γ rays viz., 50Gy (G1), 100Gy (G2), 150Gy (G3), and 200Gy (G4) using Gamma chamber Model-GC-5000 with a radioisotope Cobalt-60 at Indian Agricultural Research Institute (IARI) Pusa Campus, New Delhi, India. After the literature review, we kept the final dose as 200Gy in gamma rays and 0.4%SA in sodium azide. Before SA treatment, seeds were pre-soaked in distilled water for 9 h and then treated with SA at different doses, viz., 0.1 % (S1), 0.2 % (S2), 0.3 % (S3), and 0.4 % (S4), for 6 h under standard conditions of Mutation Breeding Laboratory, Botany Department, Aligarh Muslim University, Aligarh. In combination treatments, four gamma-irradiated seed sets comprising 300 seeds each were treated different SA doses (i.e., 50Gy γ rays+0.1 % SA (G1S1), 100Gy γ rays+0.2 % SA(G2S2), 150Gy γ rays+0.3 % SA(G3S3) and 200Gy γ rays+0.4 % SA(G4S4)) (Supplementary Tables 2–3). Two sets comprising 300 seeds each per variety were not treated with any mutagen dose and were designated a control.

### 2.3 Evaluation in M_1_ generation

In the first week of November 2016, 30 replications of treated and control seeds were sown at 60 cm inter-row, and 30 cm intra-row were sown in the randomized complete block design in an agriculture farm of Aligarh Muslim University (AMU), Aligarh (Supplementary Fig. 1). Seeds were sown in 30 replicates of 10 seeds each in both the varieties. Recommended agronomic practices such as irrigation, de-weeding, and fertilizers were followed in raising M_1_ generation. All the M_1_ plants were harvested in the first week of April 2017, and seeds were stored for raising subsequent generations.

### 2.4 Evaluation in M_2_ generation

In the first week of November 2017, 10 seeds of each M_1_ plant were sown in plant progeny rows to raise M_2_ generation. Similar agronomic practices as in the M_1_ generation were followed in raising the M_2_ generation. Screening of high-yielding plants was also carried out in the M_2_ generation. Harvesting was conducted in the first week of April 2018 and seeds were stored to raise subsequent generations.

### 2.5 Germination percentage

The seed germination percent was determined based on the total number of seeds sown in the field (Supplementary Fig. 2).

$$\text{Germination}\;\left(\%\right)=\frac{\text{No}.\;\text{of}\;\text{seeds}\;\text{germinated}}{\text{Total}\;\text{no}.\;\text{of}\;\text{seeds}\;\text{sown}\;}\times 100$$

### 2.6 Seedling height

After 15 DAS (days of sowing) in the field, seedling height was measured as a sum of the root and shoot length.

### 2.7 Seedling injury

The seedling injury was calculated as a reduction in the seedling height in mutagen-treated seedlings compared to control. The following formula is used to calculate seedling injury.

$$\text{Percentage}\;\text{injury}=\frac{\;\text{Control}-\;\text{Treated}}{\text{Control}}\;x\;100$$

### 2.8 Pollen fertility

At the flowering time, 45 plants were chosen randomly from all treatments and control to determine pollen fertility. Pollen grains were stained by acetocarmine (1 %) on glass slides covered by coverslips. Fertile pollen grains were stained, and sterile pollen grains remained unstained.

### 2.9 Estimation of carbonic anhydrase activity (CAA)

Secondary leaflets of the seedlings were collected for the estimation of CAA. Leaves weighing 200 g were washed with distilled water, chopped into small pieces, and kept in 10 mL of 0.2 M of cysteine in the Petri plates for 20 min at 4 ^o^C. The leaves were removed from the Petri plates and dried in blotting papers to remove the adhering solution. After that, these samples were poured into a new sterilized test tube containing 4 ml of phosphate buffer solution (pH 6.8). Then, 4 ml of 0.2 M sodium bicarbonate in 0.2 M sodium hydroxide solution and 0.2 ml of bromothymol blue (0.002 %) indicator were added to the samples. The test tube was put on a shaker for 20 min at 4 °C. The reaction mixture was titrated against 0.01 N hydrochloric acid using methyl red as an indicator. The titration of the control reaction mixture against 0.01 N HCL was also completed. Simultaneously, the quantity of hydrochloride used to neutralize was noted for each case, and the difference was calculated. The following formula was used to calculate the enzyme activity:

$$\text{CAA}=\frac{V\times 22\times N}{W}$$

Where.

V = Difference in the volume of HCl in the control and the sample
22 = equivalent weight of CO_2_ (liberated by the catalytic action of CAA on NaHCO₃)
N= Normality of HCl
W= Weight of tissue used

### 2.10 Chlorophyll and carotenoid assay

Secondary leaflets of the seedlings were collected to estimate pigment contents following the method of Mackinney [40]. Leaves weighing 1 g were crushed using a mortar and pestle in 80 % acetone (20 ml). The mixture was collected in a centrifuge tube and centrifuged at 5000 rpm for 5 min. After centrifugation, the supernatant was collected in a 100 ml flask. The residue was washed with 80 % acetone three times, and every wash was stored within the same volumetric flask. The final volume was made of 100 ml by adding 80 % acetone. The OD was read at 645 and 663 nm for chlorophyll and 480 and 510 nm for carotenoids. The total pigment contents were calculated using the following formula [41].

$$\text{Total}\;\text{chlorophyll}\;\left(\text{mg}.g^{‐1}\;\text{leaf}\;\text{fresh}\;\text{mass}\right)=\left\{20.2\;\left({\text{OD}}_{645}\right)+8.02\left({\text{OD}}_{663}\right)\right\}\times \frac{V}{1000\;x\;W}$$

$$\text{Carotenoid}\;\left(\text{mg}.g^{-1\;}\text{leaf}\;\text{fresh}\;\text{mass}\right)=\frac{7.6\;\left({\text{OD}}_{480}\right)-1.49\;\left({\text{OD}}_{510}\right)}{d\times 1000\times W}\times V$$

Where,

OD _645_, OD _663_, OD _480_, OD _510_ = Optical densities at OD_645,_ OD _663,_ OD_480_ and OD _510_ respectively.

V = Volume of an extract
W = Mass of leaf tissues
d = Length of light path (d = 1.4 cm)

### 2.11 Morphology parameters

In the present study, data was collected from thirty randomly selected plants in M_1_ and M_2_ generations; mean length from base to the tip represented plant height, days to flowering were measured by counting the number of days from sowing to first flowering, days to maturity was measured by counting the days from sowing to harvesting, the mean number of branches and capsules were recorded. The number of seeds per capsule was calculated by counting the seeds in ten capsules per plant randomly chosen from thirty plants. The mean 1000 seed weight was recorded by weighing the 1000 seeds, and yield per plant was calculated by measuring the total weight of seeds. The harvest index was computed by taking the ratio of seed yield and biological yield (calculated by weighing the dried weight of above-ground plant parts except for capsules) (Supplementary Table 4).

### 2.12 Genetic parameters

#### 2.12.1 Genotypic coefficient of variation (GCV)

$$\text{GCV}\;\left(\%\right)=\frac{\sqrt{\sigma^{2}g}}{\bar{x}}X\;100$$

***σ***^2^g is genotypic variance and was calculated as follows:

$$\sigma^{2}g=\frac{\left({\text{MS}}_{\text{Bf}}\;-\;{\text{MS}}_{e}\right)}{N}$$

MS_Bf_ and MS_e_ are the mean sum of squares between families and within families or error, respectively.

#### 2.12.2 Heritability (h^2^)

The broad-sense heritability (h^2^) was estimated using the following formula:

$$h^{2}=\frac{\sigma^{2}g}{\sigma^{2}p}\;X\;100$$

N = Number of replications.
***σ***^2^p is phenotypic variance and was calculated as follows:

$$\sigma^{2}p=\sigma_{g+}^{2}\sigma_{e\;}^{2}$$

***σ***^2^e is environmental variance (M_Se_ or ***σ***^2^e)

#### 2.12.3 Genetic advance (GA)

The genetic advance (GA) was calculated using the formula [42].

$$\text{GA}=k.\;\sigma p.h^{2}$$

Where.

h^2^ = Broad-sense heritability
σp = Phenotypic standard deviation of the mean performance of the treated population
K = 2.64, constant for 1 % selection intensity

#### 2.12.4 Genetic advance (percentage of mean)

$$\text{GA}\;\left(\%\;\text{of}\bar{\;X}\right)=\frac{\text{GA}}{\bar{x}}X\;100$$

### 2.13 Correlation analysis

The Spearman's correlation coefficients were calculated to check the relationship between different yield and yield attributing traits using Performance Analytics and Hmisc R packages.

## 3 Results

### 3.1 Seed germination

The results on seed germination are published in earlier publications that show a progressive decline in seed germination with an increase in mutagen doses [43].

### 3.2 Seedling height

**Fig. 1:**
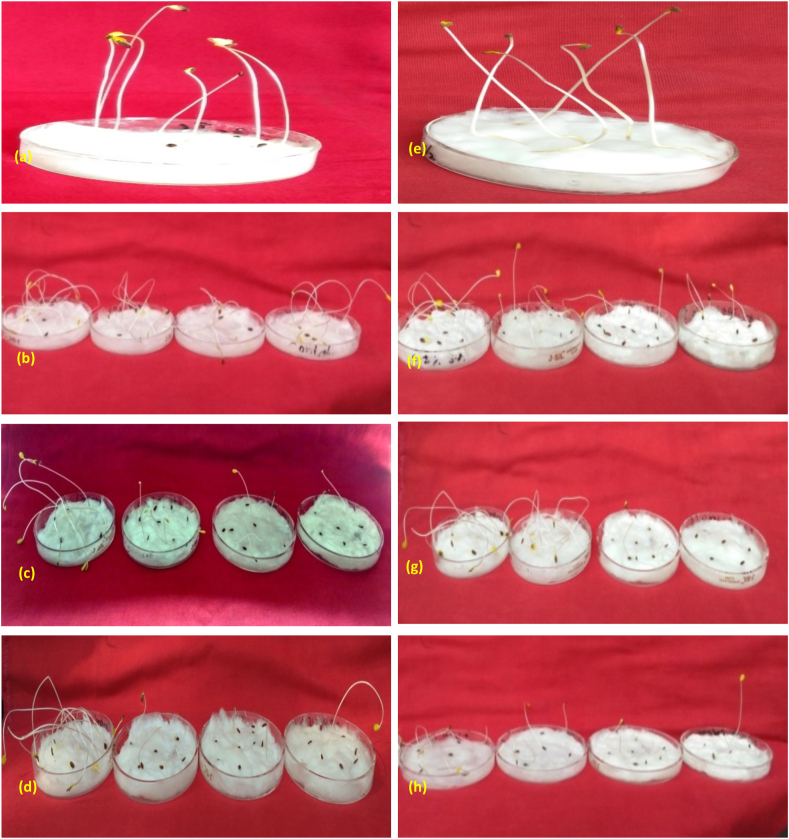
Seed germination and seedling height in **(a)** control of var. Padmini **(b)** Gamma rays treated population, lower (left) to higher dose (right), **(c)** Sodium azide treated population, lower (left) to higher dose (right), **(d)** Combination treatment, lower (left) to higher dose (right). (**e**) control of var. IC0096650. (f) Gamma rays treated population of var. IC0096650. (g) Sodium azide treated population of var. IC0096650. (h) Combination treatment of var. IC0096650.

**Fig. 2:**
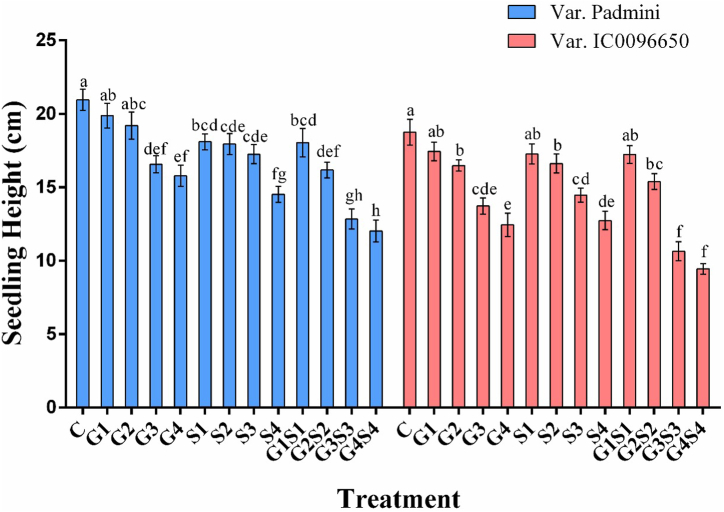
Effects of different doses of gamma rays and sodium azide on seedling height in the linseed var. Padmini and IC0096650 (n = 30). The data is presented as percent and standard error. Line graphs with the same letters are not significant at a 5 % level of significance, based on Duncan's Multiple Range Test (DMRT).

A progressive decrease in seedling height was recorded with increased mutagen doses (Fig. 1a–h). However, the highest decrease in seedling height was noted in combined mutagen treatments, followed by gamma rays and SA (Fig. 2).

### 3.3 Seedling injury

In var. Padmini, the seedling injury ranged from 5.25 to 26.05 % in 50Gy–200Gy γ rays, 11.89–31.17 % in 0.1 % SA to 0.4 % SA, and 14.21–42.53 % in 50Gy + 0.1 % SA to 200Gy + 0.4 % SA, while in the var. IC0096650, seedling injury ranged from 6.92 to 33.51 % in 50Gy–200Gy γ rays, 8.20–32.07 % in 0.1 % SA to 0.4 % SA and, 8.41–50.13 % in 50Gy + 0.1 % SA to 200Gy + 0.4 % SA treatment.

### 3.4 Cotyledonary leaf abnormalities

**Fig. 3:**
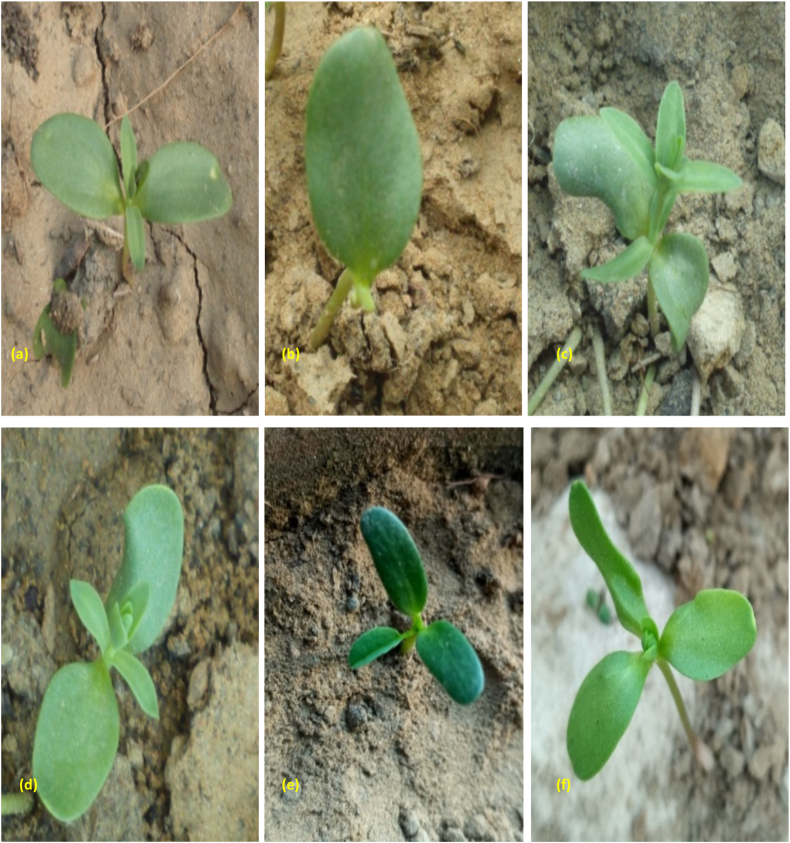
Seedlings of control and treated population with varied shape and number of cotyledonary leaves. **(a)** Seedlings with a normal pair of cotyledonary leaves (control), **(b)** Seedlings with one cotyledonary leaf, (**c)** Curved cotyledonary leaves, **(d)** Broad cotyledonary leaves, **(e)** Small, dark green tricotyledonary leaves, **(f)** Seedlings with tricotyledonary leaves. (For interpretation of the references to colour in this figure legend, the reader is referred to the Web version of this article.)

Different kinds of abnormalities in cotyledonary leaves, including curled, curved, small, large, mono-cotyledonary, and tri-cotyledonary leaves, were recorded in treated populations (Fig. 3a–f). The results revealed the highest cotyledonary leaf abnormalities in 150Gy γ rays, followed by 0.3 % SA and 150Gy γ rays+0.3 % SA in the varieties Padmini and IC0096650, respectively (Table 1, Table 2).

**Table 1:** Number and frequency of cotyledonary leaf abnormalities in the linseed var. Padmini.

Treatments	Total no. of M_1_ seedlings	Mean ± SE					Total no. of abnormal cotyledonary leaves	Total Frequency (%)
		Small cotyledonary	Large cotyledonary	Mono cotyledonary	Tri cotyledonary	Curved cotyledonary	–	–
Control	294.00	–	–	–	–	–		
50Gy γ rays	284.00	0.70^d^ ± 0.10	0.00^f^±0.00	0.35^d^ ± 0.14	0.00^d^ ± 0.00	0.35^c^±0.13	4	1.40
100Gy γ rays	277.00	0.00^g^ ± 0.00	0.36^e^±0.12	0.72^c^±0.13	0.72^b^ ± 0.15	0.00^d^ ± 0.00	5	1.80
150Gy γ rays	258.00	0.38^f^±0.13	0.77^d^ ± 0.13	0.00^e^±0.00	0.38^c^±0.11	0.77^b^ ± 0.17	6	2.32
200Gy γ rays	241.00	0.82^ab^ ± 0.13	0.00^f^±0.00	0.00^e^±0.00	0.41^c^±0.12	0.00^d^ ± 0.00	3	1.23
0.1 % SA	276.00	0.40^ef^±0.10	0.00^f^±0.00	0.40^d^ ± 0.11	0.00^d^ ± 0.00	0.40^c^±0.11	3	1.21
0.2 % SA	271.00	0.00^g^ ± 0.00	0.73^d^ ± 0.09	0.36^d^ ± 0.12	0.00^d^ ± 0.00	0.36^c^±0.14	4	1.47
0.3 % SA	261.00	0.36^f^±0.10	0.36^e^±0.13	0.36^d^ ± 0.10	0.00^d^ ± 0.00	0.72^b^ ± 0.16	5	1.80
0.4 % SA	247.00	0.38^f^±0.14	0.00^f^±0.00	0.00^e^±0.00	0.38^c^±0.14	0.38^c^±0.13	3	1.14
50Gy γ rays+0.1 % SA	270.00	0.77^bc^±0.11	1.16^b^ ± 0.17	0.38^d^ ± 0.14	0.77^b^ ± 0.11	0.00^d^ ± 0.00	8	3.11
100Gy γ rays+0.2 % SA	257.00	0.88^a^±0.14	1.76^a^±0.14	0.88^b^ ± 0.12	0.44^c^±0.12	0.44^c^±0.13	10	4.42
150Gy γ rays+0.3 % SA	226.00	0.45^e^±0.09	0.91^c^±0.20	1.36^a^±0.20	0.91^a^±0.17	1.82^a^±0.16	12	5.47
200Gy γ rays+0.4 % SA	219.00	0.74^cd^ ± 0.11	0.37^e^±0.13	0.37^d^ ± 0.12	0.74^b^ ± 0.15	0.37^c^±0.14	7	2.59

Mean within columns followed by the same letter is not different at the 5 % level of significance based on Duncan Multiple Range Test.

**Table 2:** Number and frequency of cotyledonary leaf abnormalities in the linseed var. IC0096650.

Treatments	Total no. of M_1_ seedlings	Mean ± S. E.					Total no. of abnormal cotyledonary leaves	Total Frequency (%)
		Small cotyledonary	Large cotyledonary	Mono cotyledonary	Tri cotyledonary	Curved cotyledonary	–	–
Control	289.00	–	–	–	–	–		
50Gy γ rays	279.00	0.36^c^±0.12	0.36^cd^ ± 0.14	0.00^e^±0.00	0.00^e^±0.00	0.36^c^±0.14	3	1.09
100Gy γ rays	274.00	0.39^c^±0.14	0.00^e^±0.00	0.40^d^ ± 0.13	0.00^e^±0.00	0.39^c^±0.12	4	1.45
150Gy γ rays	252.00	0.00^d^ ± 0.00	0.35^d^ ± 0.13	0.71^c^±0.14	0.71^c^±0.15	0.00^d^ ± 0.00	6	2.38
200Gy γ rays	239.00	0.41^c^±0.10	0.00^e^±0.00	0.00^e^±0.00	0.42^d^ ± 0.14	0.00^d^ ± 0.00	2	0.83
0.1 % SA	271.00	0.38^c^±0.15	0.00^e^±0.00	0.38^d^ ± 0.12	0.00^e^±0.00	0.39^c^±0.15	3	1.10
0.2 % SA	266.00	0.37^c^±0.13	0.00^e^±0.00	0.00^e^±0.00	0.37^d^ ± 0.16	0.75^b^ ± 0.15	4	1.50
0.3 % SA	259.00	0.00^d^ ± 0.00	0.36^cd^ ± 0.11	0.73^c^±0.13	0.36^d^ ± 0.15	0.37^c^±0.16	5	1.93
0.4 % SA	236.00	0.00^d^ ± 0.00	0.42^bcd^ ± 0.13	0.00^e^±0.00	0.42^d^ ± 0.14	0.00^d^ ± 0.00	2	0.84
50Gy γ rays+0.1 % SA	259.00	1.20^a^±0.17	0.80^a^±0.15	0.40^d^ ± 0.09	0.00^e^±0.00	0.40^c^±0.14	7	2.70
100Gy γ rays+0.2 % SA	249.00	0.90^b^ ± 0.18	0.44^bc^±0.14	0.89^b^ ± 0.16	1.33^a^±0.16	0.44^c^±0.14	10	4.01
150Gy γ rays+0.3 % SA	224.00	0.47^c^±0.16	0.47^b^ ± 0.13	1.42^a^±0.15	0.95^b^ ± 0.14	1.42^a^±0.16	11	4.91
200Gy γ rays+0.4 % SA	210.00	0.38^c^±0.14	0.77^a^±0.14	0.38^d^ ± 0.14	0.77^c^±0.15	0.00^d^ ± 0.00	5	2.38

Mean within columns followed by the same letter is not different at the 5 % level of significance based on Duncan Multiple Range Test.

### 3.5 Pollen fertility

**Fig. 4:**
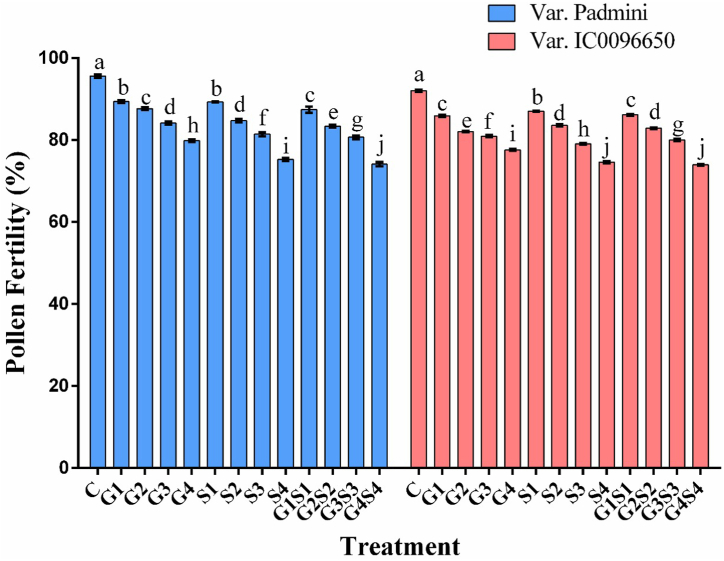
Effects of different doses of gamma rays and sodium azide on pollen fertility in the linseed var. Padmini and IC0096650 (n = 30). The data is presented as percent and standard error. Line graphs with the same letters are not significant at a 5 % level of significance, based on Duncan's Multiple Range Test (DMRT).

Pollen fertility is one of the most reliable indicators for determining mutagenic potency and genotypic sensitivity. In the treated population, a linear decrease in pollen fertility with increasing mutagen doses were recorded in both varieties (Fig. 4). However, combined mutagen doses induced a higher reduction in pollen fertility. Pollen fertility was recorded as 95.60 and 92.00 % in control populations of the varieties Padmini and IC0096650, respectively. The maximum pollen fertility was recorded in 50Gy γ rays and 0.1 % SA treatments in Padmini (89.36 %) and IC0096650 (87.02 %), respectively.

### 3.6 Carbonic anhydrase activity, chlorophyll, and carotenoid contents

**Fig. 5:**
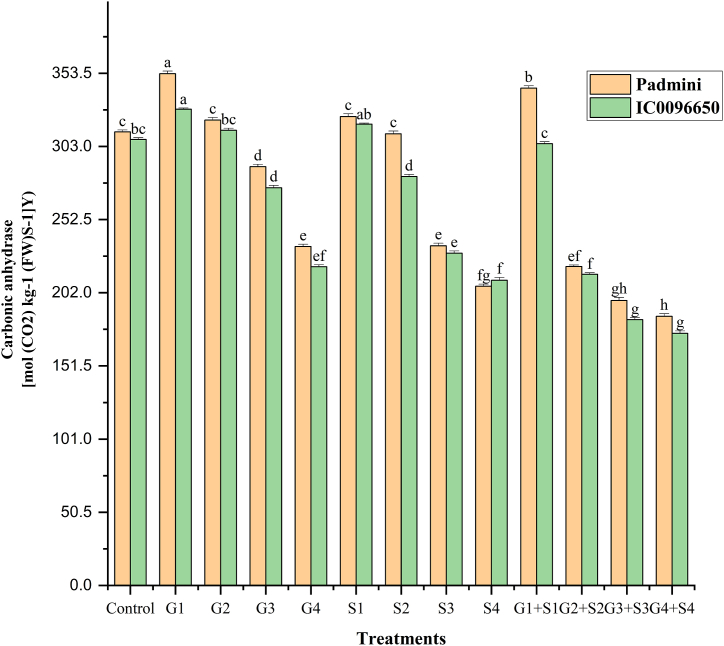
Effects of gamma rays, SA and their combinations on carbonic anhydrase activity (CAA) in linseed varieties (n = 30). The data is presented as percent (n = 30), and standard error (SE). Bars with the same letter are not different at the 5 % significance level, based on the Duncan Multiple range test.

**Fig. 6:**
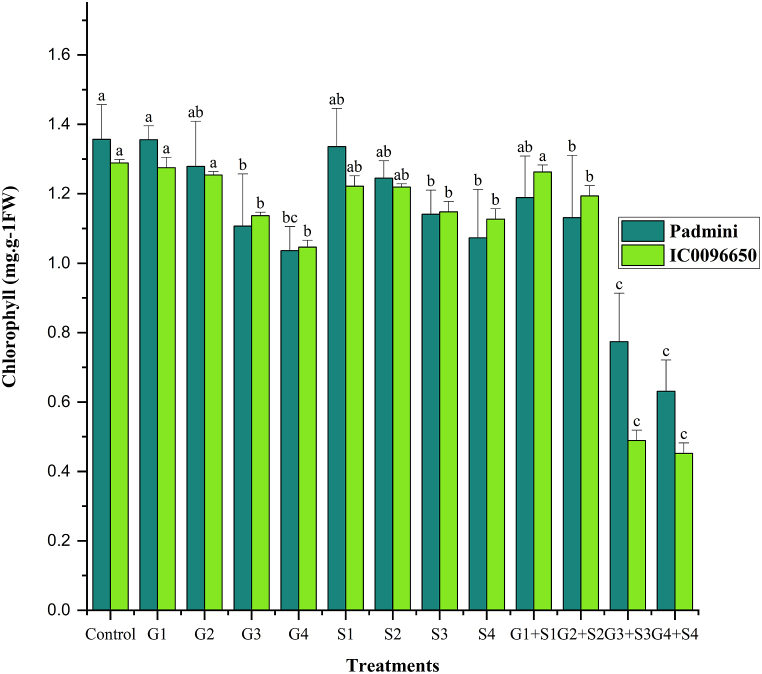
Effects of gamma rays, SA and their combinations on chlorophyll contents in linseed varieties (n = 30). The data is presented as percent (n = 30), and standard error (SE). Bars with the same letter are not different at the 5 % significance level, based on the Duncan Multiple range test.

**Fig. 7:**
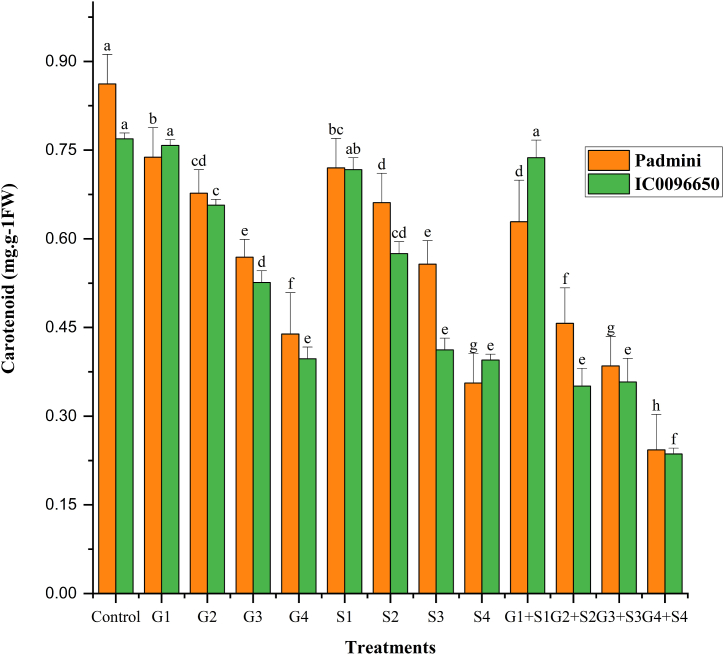
Effects of gamma rays, SA and their combinations on carotenoid contents in varieties of linseed (n = 30). The data is presented as percent (n = 30), and standard error (SE). Bars with the same letter are not different at the 5 % significance level, based on the Duncan Multiple range test.

In the present study, combination mutagen treatments revealed a drastic reduction in CAA, chlorophyll, and carotenoid contents compared to the single treatments. Overall, the CAA, chlorophyll, and carotenoid contents were higher in var. Padmini than var. IC0096650 (Fig. 5). The values of CAA were recorded as 313.01 and 307.90 mol (CO_2_) kg^−1^ (FW) S^−1^ in controls of var. Padmini and var. IC0096650, respectively (Fig. 5). The results revealed a proportionate increase and decrease in CAA activities in lower and higher mutagen treatments. The maximum CAA was recorded in 50Gy in the var. Padmini (353.23 mol (CO_2_) kg^−1^ (FW) S^−1^) and var. IC0096650 (318.41 mol (CO_2_) kg^−1^ (FW) S^−1^) (Fig. 5). In the control population, a mean chlorophyll was recorded as 1.357 and 1.289 mg g^−1^ FW in the varieties Padmini and IC0096650, respectively (Fig. 6). The results revealed a considerable decrease in the mean chlorophyll content with the minimum decrease in 200Gy + 0.4 % SA in the var. Padmini (0.631 mg g^−1^ FW) and var. IC0096650 (0.452 mg g^−1^ FW). The mean value of carotenoid content was recorded as 0.862 and 0.769 mg g^−1^ FW in controls of var. Padmini and var. IC0096650, respectively (Fig. 7). The results showed a linear reduction in mean carotenoid content with increased mutagen doses. The minimum carotenoid content was recorded in 200Gy + 0.4 % SA in var. Padmini (0.243 mg g^−1^ FW) and var. IC0096650 (0.236 mg g^−1^ FW).

### 3.7 Quantitative traits in M_1_ generation

The data collected on nine quantitative traits included plant height (cm), the number of fertile branches per plant, days to flowering, days to maturity, capsule per plant, seeds per capsule, 1000 seed weight (g), yield per plant (g) and harvest index (%) in γ rays, SA and combination treatments of both the varieties (Table 3, Table 4). One-way ANOVA showed statistically significant differences between treatments in most quantitative traits with a p-value less than 0.05. Duncan Multiple Range Test (DMRT) showed that the mean values of most quantitative traits of the treatment groups deviated significantly from the control values. The results showed a desirable shift in mean values of yield contributing traits except plant height, days to flowering and maturity, and 1000 seed weight. The mean plant height in the control populations was 74.54 and 72.94 cm in the varieties Padmini and IC0096650, respectively. In the M_1_ generation, the results revealed a considerable decrease in mean plant height with a minimum reduction in 0.3 % SA (73.24 cm) and 0.1 % SA (72.08 cm) in the varieties Padmini and IC0096650, respectively. The mean number of fertile branches per plant was 8.00 and 6.80 in control populations of varieties Padmini and IC0096650, respectively. A substantial increase in the mean number of fertile branches per plant was recorded in most mutagenic treatments, with the highest increase in 100Gy γ rays and 50Gy γ rays+0.1%SA treatment in the varieties Padmini (10.60) and IC0096650 (10.00), respectively.

**Table 3:** Estimates of mean values, and standard error for various quantitative traits of linseed var. Padmini (n = 30) in M_1_ generation.

Treatments	Plant height (cm)	Number of fertile branches/plant	Days to flowering	Days to maturity	Capsules/plant	Seeds/capsule	1000 seed weight (g)	Yield per plant (g)	Harvest index (%)
Control	74.54^a^± 0.47	8.00^cd^ ± 0.57	90.80^a^±0.80	147.40^a^±0.83	71.40^c^±0.61	9.40^d^ ± 061	7.25^cd^ ± 0.23	4.86^i^±0.20	28.82^f^±0.19
50Gy γ rays	73.14^abc^±0.69	10.40^a^±0.61	81.20^bc^±0.81	143.21^de^ ± 0.80	76.20^ab^ ± 0.80	13.60^a^±0.89	7.72^a^±0.30	8.00^a^±0.26	36.98^bc^±0.66
100Gy γ rays	71.84^cd^ ± 0.68	10.60^a^±0.66	80.60^c^±0.87	143.60^cd^ ± 0.95	76.00^ab^ ± 0.63	12.80^ab^ ± 0.80	7.65^ab^ ± 0.24	7.44^b^ ± 0.21	35.45^cd^ ± 1.02
150Gy γ rays	71.52^cde^ ± 0.50	9.20^bc^ ±0.52	82.60^b^ ± 0.71	143.80^cd^ ± 0.94	73.81^bc^±0.80	11.00^bc^±0.72	7.28^cd^ ± 0.29	5.91^f^±0.22	34.59^d^ ± 0.63
200Gy γ rays	70.54^ef^±0.65	8.40^bc^±0.62	80.20^cd^ ± 0.80	145.40^ab^ ± 0.77	69.60^cd^ ± 0.61	10.60^bcd^ ± 0.71	7.16^d^ ± 0.25	5.28^h^ ± 0.17	30.42^e^±0.52
0.1 % SA	72.52^bc^±0.52	9.60^ab^ ± 0.61	81.80^bc^±0.81	144.41^bc^±0.78	74.80^ab^ ± 0.90	13.00^a^±0.73	7.81^a^±0.28	5.59^g^ ± 0.27	38.10^a^±0.71
0.2 % SA	72.16^bcd^ ± 0.66	10.20^a^±0.65	80.40^c^±0.77	144.80^abc^±0.77	76.00^ab^ ± 0.72	12.40^ab^ ± 0.83	7.68^a^±0.23	7.23^c^±0.16	37.17^ab^ ± 0.72
0.3 % SA	73.24^ab^ ± 0.64	9.40^b^ ± 0.62	80.20^cd^ ± 0.91	146.21^ab^ ± 0.89	74.21^b^ ± 0.78	10.21^cd^ ± 0.65	7.43^bc^±0.24	5.62^g^ ± 0.30	35.66^cd^ ± 0.65
0.4 % SA	71.16^de^ ± 0.54	8.20^bcd^ ± 0.52	80.60^cd^ ± 0.78	144.00^bcd^ ± 0.72	71.61^c^±0.61	9.60^d^ ± 0.77	6.34^e^±0.22	4.35^j^±0.21	31.47^e^±0.73
50Gy γ rays+0.1 % SA	71.96^cd^ ± 0.68	10.60^a^±0.61	81.20^bc^±0.90	144.20^bcd^ ± 0.80	75.20^ab^ ± 0.80	12.40^ab^ ± 0.83	7.32^c^±0.21	6.82^d^ ± 0.23	36.42^bc^±0.76
100Gy γ rays+0.2 % SA	70.56^ef^±0.65	10.20^a^±0.52	80.60^c^±0.89	143.22^cd^ ± 0.81	76.80^a^±0.70	11.40^bc^±071	7.24^cd^ ± 0.23	6.33^e^±0.21	36.25^bc^±0.66
150Gy γ rays+0.3 % SA	69.74^fg^ ± 0.60	8.20^bcd^ ± 0.53	81.00^c^±0.72	142.40^de^ ± 0.77	74.00^b^ ± 0.72	9.41^d^ ± 0.83	7.11d ± 0.17	4.95^i^±0.22	34.27^d^ ± 0.74
200Gy γ rays+0.4 % SA	68.84^g^ ± 0.61	7.20^d^ ± 0.52	79.40^d^ ± 0.83	141.41^e^±0.87	70.41^c^±0.75	9.00^de^ ± 0.72	6.32e±0.20	4.00^k^±0.19	28.80^f^±0.50

Mean within columns followed by the same letter is not different at the 5 % level of significance based on Duncan's Multiple Range Test.

**Table 4:** Estimates of mean values, and standard error for various quantitative traits of linseed var. IC0096650 (n = 30) in M_1_ generation.

Treatments	Plant height (cm)	Number of fertile branches/plant	Days to flowering	Days to maturity	Capsule/plant	Seeds/capsule	1000 seed weight (g)	Yield per plant (g)	Harvest index (%)
Control	72.94^a^±0.55	6.80^de^ ± 0.52	95.00^a^±0.72	155.80^a^±0.80	65.80^b^ ± 0.80	8.40^ab^ ± 0.61	6.65^c^±0.23	3.67^e^±0.21	29.98^ef^±0.36
50Gy γ rays	71.84^ab^ ± 0.68	9.40^a^±0.74	88.40^bc^±0.83	153.21^abc^±0.90	69.41^a^±0.89	10.40^a^±0.71	7.18^ab^ ± 0.24	5.18^a^±0.23	34.84^ab^ ± 0.42
100Gy γ rays	71.18^bc^±0.61	8.81^ab^ ± 0.65	89.41^b^ ± 0.95	152.60^bc^±0.77	68.80^a^±0.97	10.20^a^±0.80	7.00^b^ ± 0.21	4.91^b^ ± 0.24	34.52^abc^±0.48
150Gy γ rays	69.64^cd^ ± 0.68	7.40^bcd^ ± 0.61	88.20^bc^±0.97	153.80^ab^ ±0 .80	67.60^ab^ ± 0.87	9.40^ab^ ± 0.74	6.63^c^±0.23	4.21^d^ ± 0.22	33.72^cd^ ± 0.31
200Gy γ rays	67.98^e^±0.58	7.20^cde^ ± 0.65	87.21^bc^±0.90	154.81^ab^ ± 0.81	65.81^b^ ± 0.92	9.20^ab^ ± 0.65	6.16^d^ ± 0.16	3.72^e^±0.21	29.63^ef^±0.37
0.1 % SA	72.08^ab^ ± 0.57	8.61^abc^±0.62	87.40^bc^±0.93	152.80^bc^±0.92	69.00^a^±0.95	10.40^a^±0.83	7.23^a^±0.20	5.18^a^±0.24	34.16^bc^±0.38
0.2 % SA	71.22^bc^±0.56	8.21^abc^d±0.70	86.60^bc^±0.83	153.00^bc^±0.72	67.00^ab^ ± 0.92	10.00^a^±0.72	7.13^ab^ ± 0.15	4.77^bc^±0.22	35.32^a^±0.27
0.3 % SA	69.96^cd^ ± 0.60	7.20^cde^ ± 0.71	88.00^bc^±0.78	153.40^abc^±0.83	64.60^bc^±0.97	8.80^b^ ± 0.65	6.56^c^±0.21	3.73^e^±0.24	33.11^d^ ± 0.37
0.4 % SA	68.00^e^±0.60	6.61^e^±0.61	89.41^b^ ± 0.94	153.20^abc^±0.80	62.41^c^±0.83	8.21^ab^ ± 0.65	6.00^e^±0.22	3.07^f^±0.22	30.10^e^±0.41
50Gy γ rays+0.1 % SA	70.12^cd^ ± 0.62	10.00^a^±0.72	87.20^bc^±0.80	152.81^bc^±0.93	69.21^a^±0.90	10.00^a^±0.84	7.10^ab^ ± 0.24	4.91^b^ ± 0.25	33.19^d^ ± 0.42
100Gy γ rays+0.2 % SA	69.06^de^ ± 062	9.20^ab^ ± 0.77	87.81^bc^±0.93	153.00^bc^±0.86	68.40^a^±0.94	9.61^ab^ ± 0.72	6.98^b^ ± 0.22	4.58^c^±0.22	32.98^d^ ± 0.44
150Gy γ rays+0.3 % SA	66.24^f^±0.57	7.20^cde^ ± 0.65	85.61^c^±0.75	151.80^bc^±0.92	66.00^b^ ± 0.72	8.80^ab^ ± 0.65	6.29^d^ ± 0.20	3.65^e^±0.20	29.11^fg^ ± 0.35
200Gy γ rays+0.4 % SA	64.96^f^±0.63	6.40^e^±0.62	85.80^c^±0.91	150.20^c^±0.92	64.40^bc^±0.87	7.40^b^ ± 0.61	5.98^e^±0.22	2.84^g^ ± 0.23	28.29^g^ ± 0.39

Mean within columns followed by the same letter is not different at the 5 % level of significance based on Duncan's Multiple Range Test.

In the M_1_ generation, the results showed a considerable decrease in mean days to flowering in all mutagenized plants over the control population with a maximum reduction in 200Gy γ rays+0.4 % SA and 150Gy γ rays+0.3 % SA treatment in the varieties Padmini (79.40) and IC0096650 (85.61), respectively. The results also revealed a considerable decrease in mean number of days to maturity in all mutagenized plants over the control population with a minimum reduction in 0.3 % SA and 200Gy γ rays treatments in the varieties Padmini (146.21) and IC0096650 (154.81), respectively. The results revealed a proportionate increase and decrease in the mean number of capsules per plant at lower and higher doses of γ rays and SA. The maximum increase in the mean number of capsules per plant was recorded in 100Gy γ rays+0.2 % SA and 50Gy γ rays treatments in Padmini (76.80) and IC0096650 (69.41), respectively. In control, the mean number of seeds per capsule was recorded as 9.40 and 8.40 in the varieties Padmini and IC0096650, respectively. The highest increase in mean seeds per capsule was noted in 50Gy γ rays treatment in the varieties Padmini (13.60) and IC0096650 (10.40), respectively.

The results showed a slight improvement in mean 1000 seed weight at lower and moderate doses of γ rays and SA in both varieties. The highest increase in mean 1000 seed weight was recorded in 0.1 % SA treatments in Padmini (7.81g) and IC0096650 (7.23g). The results revealed a considerable increase in mean yield per plant over the control populations in both varieties, with a maximum increase in mean yield per plant in 50Gy γ rays treatments in the varieties Padmini (8.00g) and IC0096650 (5.18g), respectively. The results also revealed a considerable increase in harvest index in all mutagenized populations with a maximum increase in 0.1 % SA and 0.2 % SA treatments in the varieties Padmini (38.10) and IC0096650 (35.32), respectively.

### 3.8 Quantitative traits in M_2_ generation

In the M_2_ generation, we collected data on the quantitative traits in a similar pattern to that of the M_1_ generation (Table 5, Table 6). The results revealed that mean days to flowering in the treated population shifted negatively. The maximum reduction in days to flowering was recorded in 50Gy γ rays in the var. Padmini (75.83) as compared to control (89.73). In the var. IC0096650, 50Gy treated population also showed a maximum reduction in days to flowering (85.83) compared to control (94.20). In the control, plant height was 75.71 and 72.17 cm in the varieties Padmini and IC0096650, respectively. A significant negative shift in mean values of plant height was noted in the treated population of both varieties. Maximum decline in the plant height was noted in 200Gy + 0.4 % SA in the varieties Padmini (69.23 cm) and IC0096650 (63.23 cm). In control, the maturity period was recorded as 148.06 and 155.83 days in the varieties Padmini and IC0096650, respectively. The maturity was earlier by 5.83 and 5.84 days in the varieties Padmini and IC0096650, respectively. A maximum decline in days to maturity was noted in the 200Gy + 0.4 % SA treated population of the varieties Padmini (142.23 days) and IC0096650 (150 days) in 50Gy γ rays.

**Table 5:** Estimates of mean values, ±S.E.standard error (n = 30), genotypic coefficient of variation (GCV %), broad-sense heritability (ℎ^2^ %) and genetic advance as % of the mean (GA %) for the nine quantitative traits in M_2_ generation of linseed var. Padmini.

TRAITS		TREATMENT POPULATIONS												
		C	G1	G2	G3	G4	S1	S2	S3	S4	G1+S1	G2+S2	G3+S3	G4+S4
**Plant Height (cm)**	MEAN^a^	75.71^a^± 0.28	73.67^b^ ± 0.37	72.58^bc^±0.28	72.20^bcd^ ± 0.35	70.89^cde^ ± 0.26	73.49^b^ ± 0.28	72.45^bc^±0.27	73.57^b^ ± 0.29	72.00^bcde^ ± 0.26	71.77^cd^ ± 0.25	71.21^cde^ ± 0.37	70.02^de^ ± 0.34	69.23^e^±0.34
	GCV %	1.03	3.69	2.41	2.02	1.74	3.08	3.27	2.59	1.88	3.21	2.34	1.97	1.58
	h^2^ %	21.25	77.16	64.83	55.58	47.12	69.72	70.33	67.40	53.27	69.92	65.04	58.71	59.31
	GA%	1.22	8.52	5.04	3.93	3.15	6.73	7.19	5.60	3.62	7.02	4.98	3.92	3.19
**Days to Flowering**	MEAN^a^	89.73^a^±0.61	75.83^f^ ±0.60	76.73^ef^ ±0.71	81.03b ± 0.59	80.00^bc^±0.69	78.90^bcde^ ± 0.74	78.00^cde^ ± 0.82	79.51^bcd^ ± 0.72	79.83^bcd^ ± 0.83	77.00^ef^±0.88	77.53^def^±0.72	79.73^bcd^ ± 0.75	79.06^bcde^ ± 0.70
	GCV %	1.70	4.19	5.27	3.73	3.26	3.86	3.53	2.72	2.46	3.75	5.95	2.25	2.02
	h^2^ %	32.86	62.85	69.46	58.41	56.64	61.70	51.61	48.96	41.02	59.16	68.70	37.10	33.72
	GA%	2.50	8.65	11.53	7.48	6.41	7.96	6.67	4.95	4.17	7.58	12.92	3.61	3.05
**Days to Maturity**	MEAN^a^	148.06^a^ ±0.52	143.93^def^±0.61	144.33^cde^ ± 0.75	144.16^def^±0.66	146.53^abc^±0.67	144.06def±0.61	145.56^bcde^ ± 0.71	147.53 ab ± 0.73	145.76^bcd^ ± 0.83	145.66^bcde^ ± 0.86	144.16^def^±0.79	143.36^ef^±0.83	142.23^f^±0.59
	GCV %	1.12	3.43	2.94	2.66	2.14	3.35	3.02	2.56	1.98	3.70	3.97	2.55	2.00
	h^2^ %	34.53	80.09	75.01	60.14	46.82	78.54	75.97	59.03	43.85	81.02	81.33	56.55	45.08
	GA%	1.71	8.11	6.73	5.43	3.82	7.80	6.86	5.19	3.41	8.80	9.43	5.04	3.54
**No of Fertile Branches per plant**	MEAN^a^	8.63^ef^±0.20	11.13^ab^ ± 0.38	11.53^a^ ±0.41	9.53de ± 0.26	8.96^def^±0.25	9.73^cd^ ± 0.29	10.53^bc^±0.33	9.50^de^ ± 0.35	8.53f±0.28	11.03^ab^ ± 0.34	10.73^ab^ ± 0.31	9.43^de^f±0.27	7.56^g^ ± 0.26
	GCV %	8.37	14.73	15.95	12.69	11.71	12.23	13.48	11.89	9.61	15.14	13.04	11.77	7.80
	h^2^ %	23.42	53.03	57.19	42.48	39.85	40.00	47.33	38.23	29.18	52.12	43.65	39.43	17.85
	GA%	10.31	28.39	31.83	21.61	19.08	20.40	24.39	19.43	13.59	28.83	22.27	19.43	8.20
**Capsulesper plant**	MEAN^a^	72.83^ef^±0.39	79.06^a^ ±0.54	78.73^a^ ±0.55	74.46^de^ ± 0.68	72.73^e^f±0.64	76.76^b^c±0.69	78.03^ab^ ± 0.67	75.43^cd^ ± 0.52	73.86e± 0.68	76.46^b^c±0.60	78.63^a^±0.59	75.33^c^d ± 0.57	71.83^f^ ±0.58
	GCV %	2.71	7.15	6.40	4.74	3.97	6.81	6.94	4.25	3.54	6.97	5.26	4.04	3.13
	h^2^ %	39.59	90.20	87.35	71.92	62.12	85.69	86.33	68.33	54.90	86.57	71.52	54.79	45.98
	GA%	4.43	17.19	15.75	10.47	8.25	16.51	16.96	9.25	6.81	17.01	11.68	11.68	5.49
**Seeds per Capsule**	MEAN^a^	8.87^f^±0.20	13.13^a^±0.36	12.16^b^ ± 0.31	10.43^de^ ± 0.29	9.40^f^±0.23	12.23^b^ ± 0.30	11.43^bc^±0.31	9.76^ef^±0.29	9.43^f^±0.30	12.03^b^ ± 0.31	11.06^cd^ ± 0.28	9.56^f^±0.29	9.06^f^±0.26
	GCV %	13.95	23.76	22.12	19.17	17.65	22.57	20.03	18.13	17.60	25.51	21.42	18.09	18.00
	h^2^ %	57.14	80.36	78.82	70.97	64.85	79.37	71.95	61.60	59.35	84.14	72.62	66.89	64.65
	GA%	27.83	55.97	51.31	41.99	36.84	52.69	44.27	37.12	35.51	61.75	47.77	38.45	37.85
**1000** **Seed Weight (g)**	MEAN^a^	8.00^a^±0.04	7.84^ab^ ± 0.09	7.80^abc^ ±0.07	7.53^d^ ± 0.10	7.30^e^±0.07	7.90^ab^ ± 0.06	7.72^bcd^ ± 0.07	7.55^d^ ± 0.06	6.85^f^±0.06	7.58^cd^ ± 0.05	7.54^d^ ± 0.09	7.02^f^±0.06	6.47^g^ ± 0.04
	GCV %	3.06	8.92	7.94	7.17	6.30	8.35	6.99	5.43	4.67	10.94	8.22	7.54	7.26
	h^2^ %	45.45	79.36	73.58	69.04	66.65	77.19	72.50	65.38	58.82	82.35	73.58	68.29	65.71
	GA%	5.27	21.01	17.78	15.48	13.60	19.29	15.51	11.36	9.16	26.27	18.40	16.36	15.64
**Total Yield (g)**	MEAN^a^	5.16^g^ ± 0.03	8.13^a^ ±0.06	7.46^b^ ± 0.07	5.84^e^ ±0.08	5.00^g^ ± 0.05	7.41^b^ ± 0.07	6.88^c^±0.07	5.55^f^±0.08	4.77^h^ ± 0.07	6.97^c^ ±0.05	6.56^d^ ± 0.05	5.05^g^ ± 0.06	4.21^i^ ±0.07
	GCV %	3.10	9.22	7.50	5.60	4.40	8.09	7.26	5.94	4.82	6.74	5.48	5.14	4.98
	h^2^ %	20.00	86.36	77.50	65.21	45.46	82.22	73.52	61.11	41.66	74.19	72.22	53.84	40.00
	GA%	3.17	22.62	17.16	13.81	7.84	19.57	16.24	12.18	7.71	15.41	12.16	9.97	7.77
**Harvest Index (%)**	MEAN^a^	29.27^j^±0.10	40.06^a^ ±0.19	36.84^cd^ ± 0.25	35.23^f^ ±0.27	31.17^i^ ±0.18	39.02^b^ ± 0.23	37.22^c^±0.15	35.82^ef^±0.16	32.83^h^ ± 0.20	38.57^b^ ± 0.37	36.23^de^ ± 0.13	33.92^g^ ± 0.16	30.97^i^ ±0.17
	GCV %	2.18	6.71	4.96	4.51	3.88	5.61	4.43	3.51	3.01	5.02	3.78	3.00	2.74
	h^2^ %	31.11	83.52	69.05	61.29	58.33	78.62	65.07	60.68	55.00	73.59	61.88	51.74	48.97
	GA%	3.24	16.08	10.87	9.27	7.76	13.03	9.40	7.11	5.92	11.29	7.77	5.59	4.95

a Means within rows followed by the same letter is not different at the 5 % level of significance, based on the Duncan Multiple range test.

**Table 6:** Estimates of mean values, ±S.E. standard error (n = 30), genotypic coefficient of variation (GCV %), broad sense heritability (*ℎ*^2^ %) and genetic advance as % of the mean (GA %) for the quantitative traits in M_2_ generation of linseed var. IC0096650.

TRAITS		TREATMENT POPULATIONS												
		C	G1	G2	G3	G4	S1	S2	S3	S4	G1+S1	G2+S2	G3+S3	G4+S4
**Plant Height (cm)**	MEAN^a^	72.17^a^ ±0.24	71.76^a^ ±0.36	70.10^b^ ± 0.27	68.46^de^ ± 0.26	67.85^e^±0.30	72.00^a^ ±0.32	70.19^b^ ± 0.19	69.72^bc^ ±0.31	67.69^e^ ±0.26	69.00^cd^ ± 0.26	68.25^de^ ± 0.28	65.82^f^ ±0.25	63.23^g^ ± 0.22
	GCV %	1.12	4.34	2.15	1.74	1.35	3.83	2.99	1.89	1.66	3.08	2.10	1.53	1.40
	h^2^ %	27.84	84.05	57.17	44.53	31.36	81.41	69.93	54.85	48.48	71.07	58.50	42.50	39.60
	GA%	1.51	10.51	4.29	3.10	1.97	9.05	6.51	3.63	3.03	6.87	4.19	2.59	2.31
**Days to Flowering**	MEAN^a^	94.20^a^ ±0.39	85.83^e^ ±0.49	87.00^cde^ ± 0.40	88.10^bc^ ±0.46	87.16^cde^ ± 0.56	86.96^cde^ ± 0.46	86.23^de^ ± 0.36	87.53^bcd^ ± 0.47	88.23^bc^±0.45	86.00^e^±0.43	86.26^de^ ± 0.46	88.40^bc^±0.56	89.00^b^ ± 0.59
	GCV %	1.33	3.60	2.68	2.44	2.21	3.10	2.51	2.22	1.97	3.01	2.30	1.96	1.51
	h^2^ %	28.15	64.51	59.48	46.55	30.50	60.24	62.51	39.50	34.74	61.55	42.24	40.90	24.96
	GA%	1.88	7.63	5.43	4.35	3.17	6.34	5.19	3.65	2.96	6.17	3.91	3.25	1.92
**Days to Maturity**	MEAN^a^	155.83^a^ ±0.64	150.00^e^ ±0.71	150.73^de^ ± 0.71	153.83^ab^ ± 0.78	153.96^ab^ ± 0.81	151.20^cde^ ± 0.68	152.80^bcd^ ± 0.70	153.40^bc^±0.81	153.26^bc^±0.98	152.00^bcde^ ± 0.77	153.00^bcd^ ± 0.79	154.00^ab^ ± 0.67	154.30^ab^ ± 0.59
	GCV %	1.09	3.06	2.42	1.89	1.48	2.94	2.59	1.88	1.38	3.21	2.70	2.05	1.82
	h^2^ %	36.69	66.32	55.72	46.69	41.15	65.53	59.47	40.96	36.71	68.93	61.66	55.40	52.19
	GA%	1.71	6.32	4.71	3.36	2.49	6.24	5.23	3.10	2.16	6.94	5.53	4.01	3.46
**No of Fertile Branches per plant**	MEAN^a^	6.96d^ef^±0.17	9.96^a^ ±0.26	8.96^bc^±0.24	7.43^d^ ± 0.19	7.00^def^±0.17	8.96^bc^±0.25	8.53^c^ ±0.27	7.10^de^ ± 0.18	6.46^ef^±0.17	9.03^bc^ ±0.25	9.23b ± 0.26	7.00^def^±0.21	6.33^f^±0.16
	GCV %	7.04	11.64	9.37	8.88	7.85	10.04	8.90	8.45	7.43	10.85	9.42	7.57	7.26
	h^2^ %	15.28	39.19	26.76	22.91	19.23	28.17	27.52	18.94	17.03	31.80	**27.83**	18.54	16.40
	GA%	7.18	19.22	12.56	10.78	8.89	14.02	12.28	9.16	8.05	15.76	12.74	8.28	7.54
**Capsulesperplant**	MEAN^a^	65.96^e^ ±0.44	71.36^ab^ ± 0.57	72.50^a^±0.59	70.23bcd ± 0.51	69.30^cd^ ± 0.62	71.00^abc^±0.76	71.20^ab^ ± 0.58	69.13^d^ ± 0.61	68.56d ± 0.68	71.23^ab^ ± 0.43	70.13^bcd^ ± 0.45	68.73^d^ ± 0.55	66.26^e^ ±0.44
	GCV %	1.84	4.58	4.86	3.64	2.66	3.92	3.75	2.56	2.17	4.29	3.55	2.41	2.06
	h^2^ %	33.25	67.25	69.30	59.36	47.45	61.79	60.37	49.60	41.26	64.53	56.80	42.24	38.13
	GA%	2.78	9.89	10.65	7.36	4.81	8.05	7.63	4.71	3.64	9.03	6.95	4.12	3.36
**Seeds per Capsule**	MEAN^a^	7.40^ef^ ±0.20	10.23^a^ ±0.26	10.00^a^ ±0.31	9.40^abc^±0.24	9.00^bc^±0.26	10.10^a^ ±0.27	10.20^a^ ±0.33	8.76^cd^ ± 0.23	8.13^de^ ± 0.23	10.00^a^ ±0.32	9.73^ab^ ± 0.28	8.63^cd^ ± 0.26	7.23^f^ ±0.24
	GCV %	12.94	21.01	20.20	16.38	15.77	18.21	19.80	16.89	16.35	20.40	18.60	15.99	14.52
	h^2^ %	53.27	73.11	70.51	68.67	61.58	65.83	69.38	62.53	61.88	66.71	65.53	64.86	61.45
	GA%	24.82	47.40	44.30	35.42	32.33	38.51	43.13	35.04	33.45	43.50	39.46	33.60	29.59
**1000** **Seed Weight (g)**	MEAN^a^	7.80^a^±0.04	7.52^b^ ± 0.05	7.16^e^±0.04	6.69^f^ ±0.05	6.42^g^ ± 0.04	7.32^cd^ ± 0.04	7.19^de^ ± 0.05	6.78^f^±0.06	6.31^g^ ± 0.05	7.40^bc^ ±0.06	7.10^e^ ±0.07	6.66^f^ ±0.04	6.00^h^ ± 0.06
	GCV %	2.50	4.38	3.91	3.58	3.27	4.23	4.03	3.98	3.48	5.13	4.22	3.30	3.16
	h^2^ %	37.50	64.70	61.53	55.55	44.46	75.00	66.66	46.66	45.46	71.42	64.28	50.01	42.85
	GA%	3.98	9.17	8.09	6.51	5.42	9.15	8.20	6.78	6.18	11.35	8.73	6.00	4.66
**Total Yield (g)**	MEAN^a^	3.80^f^ ±0.03	5.48^a^±0.05	5.21^b^ ± 0.07	4.41^d^ ± 0.06	4.00^e^ ±0.05	5.25^b^ ± 0.05	5.14^b^ ± 0.07	4.10^e^±0.04	3.51^g^ ± 0.05	5.27^b^ ± 0.03	4.84^c^ ±0.05	3.95^ef^±0.05	2.88^h^ ± 0.04
	GCV %	2.63	5.65	4.98	3.85	3.50	4.57	4.29	3.41	3.13	5.69	4.54	3.54	3.47
	h^2^ %	12.50	83.34	77.78	50.01	40.00	66.68	41.66	33.34	20.00	90.00	71.42	40.00	20.00
	GA%	2.10	13.50	11.51	7.02	5.75	9.90	7.00	4.87	3.30	13.85	9.92	5.88	3.81
**Harvest Index (%)**	MEAN^a^	30.83^hi^±0.12	38.24^a^ ±0.16	36.23^cd^ ± 0.17	35.18e ±0.28	31.03hi±0.41	37.45^ab^ ± 0.33	34.00^f^ ±0.48	33.45^f^ ±0.47	32.22^g^ ± 0.36	37.05^b^c±0.39	35.63^de^ ± 0.44	31.78^gh^ ± 0.37	30.29^i^±0.31
	GCV %	1.65	3.73	3.03	2.70	2.28	3.95	2.91	2.57	2.04	3.56	2.83	2.61	2.01
	h^2^ %	27.55	77.44	63.82	57.86	47.66	70.00	60.36	54.74	45.83	73.64	60.23	54.33	43.18
	GA%	2.23	8.65	6.28	5.37	4.09	9.29	5.94	4.96	3.56	7.98	5.75	5.00	3.46

a Means within rows followed by the same letter is not different at the 5 % level of significance, based on the Duncan Multiple range test.

The results revealed that mutagens substantially increased the mean values of fertile branches per plant in both varieties. The mean number of fertile branches per plant was 8.63 and 6.96 in the control population of the varieties Padmini and IC0096650, respectively. The highest increase in the mean fertile branches per plant was noted in 100Gy and 50Gy γ rays in the varieties Padmini (11.53) and IC0096650 (9.96), respectively. The highest increase in the mean capsules per plant was recorded in 50Gy and 100Gy γ rays in the varieties Padmini (79.06) and IC0096650 (72.50), respectively. The mean capsule per plant was recorded as 72.83 and 65.96 in the control population of the varieties Padmini and IC0096650, respectively. The results showed that single and combined treatments of γ rays and sodium azide were ineffective in inducing significant differences in the mean seeds per capsule. The mean seeds per capsule were recorded as 8.87 and 7.40 in the control population of the varieties Padmini and IC0096650, respectively. The mean seeds per capsule were highest (13.13 and 10.23) in 50Gy γ rays treatment in the varieties Padmini and IC0096650, respectively. All doses of γ rays, SA, and their combination treatments decreased the mean seed weight compared to the control. The mean 1000 seed weight was recorded as 8.00 and 7.80 g in the control population of the varieties Padmini and IC0096650, respectively. Minimum reduction in the mean 1000 seed weight was recorded as 7.90 and 7.52 g in 0.1 % SA and 50Gy γ rays treatments in the varieties Padmini and IC0096650, respectively. The mean seed yield per plant of the treated population differed significantly from controls of both varieties. The mean seed yield was recorded as 5.16 and 3.80 g in the control population of the varieties Padmini and IC0096650, respectively. The highest increase in yield per plant was noted as 8.13 and 5.48 g in the varieties Padmini and IC0096650, respectively. In general, the mean seed yield per plant increased more in var. Padmini than var. IC0096650. The mean values of the harvest index shifted positively and negatively in the mutagenized population. In general, the lower and intermediate doses of single and combined mutagen doses induced a positive shift in the mean values over controls of both varieties. In control, the mean harvest index was recorded as 29.27 and 30.83 in the varieties Padmini and IC0096650, respectively.

### 3.9 Genetic coefficient of variability, heritability, and genetic advance

The trait-wise results from the present study are discussed below and in Table 5, Table 6

#### 3.9.1 Days to flowering

In both the varieties, GCV, h^2^ and GA showed a manifold increase over the control. Most of the mutagenic treatments recorded an increase in heritability coupled with genetic advances. The maximum GCV (5.95 and 3.60 %) and GA (12.92 and 7.63 %) were recorded in 100Gy + 0.2 % SA and 50Gy treatments in the varieties Padmini and IC0096650, respectively. The maximum h^2^ (69.46 and 64.51 %) was recorded in 100Gy and 50Gy γ rays treatments in the varieties Padmini and IC0096650, respectively.

#### 3.9.2 Plant height

The maximum GCV (3.69, 4.34 %), h^2^ (77.16, 84.05 %), and GA (8.52, 10.51 %) were recorded in 50Gy γ rays in the varieties Padmini and IC0096650, respectively.

#### 3.9.3 Days to maturity

The maximum GCV (3.97 and 3.21 %), h^2^ (81.33, 68.93 %), and GA (9.43 and 6.94 %) were recorded in 100Gy + 0.2 % SA and 50Gy + 0.1 % SA in the varieties Padmini and IC0096650, respectively.

#### 3.9.4 Number of fertile branches per plant

The highest GCV (15.95 and 11.64 %), h^2^ (57.19 and 39.19 %), and GA (31.83 and 19.22 %) were recorded in 100Gy and 50Gy γ rays treatments in the varieties Padmini and IC0096650, respectively.

#### 3.9.5 Capsules per plant

The maximum GCV (7.15 and 4.86 %), h^2^ (90.20 and 69.30 %), and GA (17.19 and 10.65 %) were recorded in 50Gy and 100Gy γ rays treatment in the varieties Padmini and IC0096650, respectively.

#### 3.9.6 Seeds per capsule

The maximum GCV (25.51 and 21.01 %), h^2^ (84.14 and 73.11 %), and GA (61.75 and 47.40 %) were recorded in 50Gy + 0.1 % SA and 50Gy γ rays treatment in the varieties Padmini and IC0096650, respectively.

##### 3.9.6.1 1000 seed weight (g)

The maximum GCV (10.94 and 5.13 %) and GA (26.27 and 11.35 %) were recorded in 50Gy + 0.1 % SA treatment in the varieties Padmini and IC0096650, respectively. The highest h^2^ (82.35 and 75.00 %) was recorded in 50Gy + 0.1 % SA and 0.1 % SA treatments in the varieties Padmini and IC0096650, respectively.

#### 3.9.7 Seed yield per plant (g)

The genetic parameters increased in all mutagen treatments compared with control in both varieties. The highest GCV (9.22 and 5.69 %), h^2^ (86.36 and 90.00 %), and GA (22.62 and 13.85 %) were observed in 50Gy and 50Gy + 0.1 % SA treatments in the varieties Padmini and IC0096650, respectively.

#### 3.9.8 Harvest index (%)

The highest GCV (6.71 and 3.95 %), h^2^ (83.52 and 70.00 %), and GA (16.08 and 9.29 %) were recorded in 50Gy and 0.1 % SA treatment in the varieties Padmini and IC0096650, respectively.

### 3.10 Correlation analysis

**Fig. 8:**
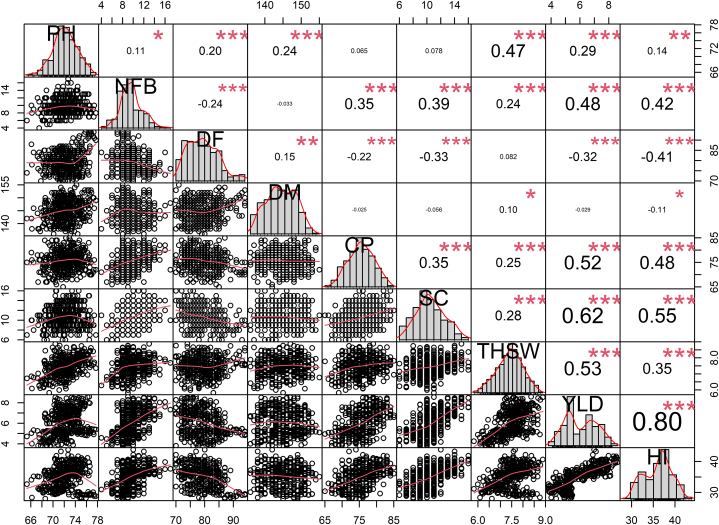
Correlation analysis between yield and yield attributing traits in variety Padmini. PH, Plant height; NFB, Number of fertile branches; DF, Days to flowering; DM, Days to maturity; CPP, Capsules per plant; SC Seeds per capsule; THSW, 1000-seed weight; YLD, Seed yield per plant; HI, Harvest index. Significant differences are indicated as ***P < 0.001, **P < 0.01, and *P < 0.05.

**Fig. 9:**
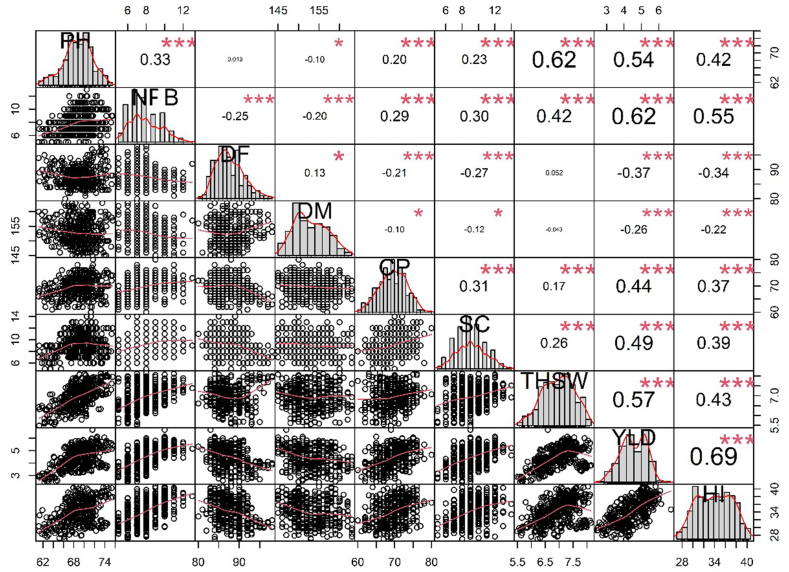
Correlation analysis between yield and yield attributing traits in variety IC0096650. PH, Plant height; NFB, Number of fertile branches; DF, Days to flowering; DM, Days to maturity; CPP, Capsules per plant; SC Seeds per capsule; THSW, 1000-seed weight; YLD, Seed yield per plant; HI, Harvest index. Significant differences are indicated as ***P < 0.001, **P < 0.01, and *P < 0.05.

The results of the present study showed a positive and significant correlation between yield and yield-attributing traits. The highest positive correlation was recorded between yield and harvest index (0.80), followed by yield and seeds per capsule (0.62) in the variety Padmini (Fig. 8). In the variety IC0096650, the maximum positive correlation was recorded between yield and harvest index (0.69) followed by yield and number of fertile branches per plant (0.62) (Fig. 9).

## 4 Discussion

### 4.1 Bio-physiological traits

The observation of different bio-physiological parameters revealed that linseed var. IC0096650 was relatively more sensitive to mutagen treatments than var. Padmini. Differences between the varieties of the same species or genus towards identical mutagens were also reported earlier in *Cicer arientinum* [44], *Lens culinaris* [23], *Vigna unguiculata* [35]*.* Moreover, the differential sensitivity of varieties toward the mutagens and mutagen doses might be attributed to substantial variations in their genetic makeup [45]. In this study, two linseed varieties, were chosen from different races; therefore, it is apparent that the two varieties possess significant variations in their genome and, hence, differ in the sensitivity toward mutagen treatments. The study of mutagen-induced bio-physiological damages is a reliable indicator in evaluating the genotypic sensitivity and potency of a particular mutagen. Biological damages induced by mutagen could be considered an indicator of mutagenic sensitivity of the genotypes [46]. In the present study, higher mutagen doses induced more reduction in bio-physiological traits that might be attributed to cellular disturbances [47]. The results were in accordance with the findings of [48] in *Cicer arietinum* and are discussed below:

#### 4.1.1 Seed germination

Synergistic effects of two mutagens might be attributed to higher seed germination inhibition noted in combination treatments of both varieties[43]. Reductions in seed germination after mutagenic treatments were earlier reported in various crops, such as *Cicer arietinum* [49], *Lens culinaris* [50], and *Vigna unguiculata* [51]. The decline in germination percentage could be due to the mutagen-induced disruptions in the genetically controlled bio-physiological and metabolic pathways that play a crucial role in the seed germination process. These processes include altered enzyme activity, inhibition of the mitotic cycle, and hormonal imbalances [50,51].

#### 4.1.2 Seedling height

The reduced seedling height in the treated population in both varieties could be attributed to mutagen-induced auxin destruction and altered enzyme activity [52]. A decrease in seedling height after mutagen treatments is mainly due to variations in auxin level and inhibition of mitotic proliferation [53]. Chromosomal damage or inhibition of cell division or alteration in enzyme activity induced by mutagens may also be one of the main reasons for reduced seedling height [54,55]. Compared to the gamma rays and sodium azide, combination treatments induced a maximum reduction in seedling height. The results were in accordance with the findings of [56,57], who reported a dose-dependent decrease in seedling height of *Trigonella foenum-graecum* and *Vigna unguiculata*, respectively.

#### 4.1.3 Cotyledonary leaves

The control seedling has two cotyledonary leaves with opposite phylotaxy. The results revealed a dose-independent increase in cotyledonary leaf abnormalities (variations in the size, shape, and number) with the highest frequency in intermediate doses of individual and combined mutagens. The results were in agreement with the earlier studies, which reported maximum frequency of cotyledonary leaf abnormalities at intermediate doses of gamma rays and EMS employed singly and in combinations [58]. Mutagen-induced abnormalities in cotyledonary leaves may be attributed to alterations in leaf primordia. Several workers also noticed similar abnormalities in crops such as *Vigna radiata* [59,60]. The emergence of a single cotyledonary leaf in a few seedlings may be attributed either to cytochemical disturbance or to acute chromosomal anomalies leading to the death of the leaf primordial or the embryonic cell, which is responsible for the development of the leaf [61]. The development of extra cotyledonary leaves demonstrates the formation and presence of additional leaf primordial or embryonic cells.

#### 4.1.4 CAA, chlorophyll, and carotenoid contents

A proportionate increase and decrease in CAA activities in lower and higher mutagen treatments may be attributed to alterations in genes associated with CA activities [62]. In the present study, a progressive decrease in chlorophyll and carotenoid contents in all mutagen-treated plants may be due to increased chlorophyllase activity and down-regulation of carotenoid biosynthesizing genes, respectively. The results were in propinquity with the earlier findings in *Trigonella foenum-graecum* [56], *Vicia faba* [63], *Eruca sativa* [64], *Satureja hortensis* [65], *Linum usitatissimum* [66] and *Eleusine coracana* [67].

#### 4.1.5 Pollen fertility

Pollen grain structure and physiology are genetically controlled, and any abnormal meiotic aberration can lead to significant variation in the properties of pollen grains as observed in the form of higher pollen sterility in combination treatments. The results were in good agreement with the earlier findings in *Vicia faba* [63], *Lens culinaris* [46], and *Linum usitatissimum* [66]. Pollen sterility may be attributed to a broad spectrum of meiotic abnormalities caused by mutagens leading to the formation of aberrant pollen grains. Meiotic abnormalities have been demonstrated to cause pollen sterility in various plants [31,68] and such reduction in pollen fertility is often correlated with meiotic abnormalities [66,69]. Earlier studies reported that pollen sterility resulted from the interchange of segments between non-homologous chromosomes [70].

### 4.2 Quantitative traits

In the present study, a shift in mean values in both positive and negative directions may be due to the role of multiple genes that govern quantitative traits. For instance, a reduction in the mean values of plant height may be attributed to mutagen-altered multiple genes that govern cellular divisions. The short-stature mutagenized plants are good at resisting the fast-moving wind prevalent during the harvest season of linseed. Some workers also found a reduction in plant height due to mutagenic treatments in lentil [50], fenugreek [56], and finger millet [67]. In the present study, increased branching in mutagenized plants is directly associated with increased yield. Mutants with more branches have been reported in earlier studies [46,50]. Few early maturing mutants with shorter growth periods isolated in the present study could escape several biotic and abiotic stresses and their detrimental effects on plant growth, development and yield. Earlier studies have also reported early maturing mutants in cowpea, tomato, and lentil [,[71], [72]]. A reduced maturity period may be due to the physiological effects of gamma rays and sodium azide. While collecting data on quantitative traits, a few mutants with increased capsules per plant and seeds per capsule were also recorded. Such mutants must be advanced for future generations at multi-location to check the trait fixation and facilitate the release of high-yielding linseed varieties. Earlier workers also reported mutants with more capsules and seeds [73]. Seed weight is a reliable index for assessing yield; any seed weight increase is directly associated with the linseed improvement. A considerable increase in 1000 seed weight and yield per plant in a few mutants reflected that mutagens were effective in inducing desirable mutations and could lead to developing valuable genetic resources in the form of high-yielding mutant lines. Many workers have also reported increased seed yield in various mutagenized crops such as *Vigna unguiculata* [35] and *Cicer arietinum* [48]. A substantial increase in the mean harvest index revealed an efficient allocation of assimilated photosynthate to the seeds. Many workers have also reported similar findings in various crop species [63,74].

### 4.3 Genetic parameters of quantitative traits

The genetic enhancement of quantitative traits depends upon the degree of genetic parameters and the methodology used for breeding. To formulate an effective breeding strategy and determine the highest genetic improvement for a specific trait that can be obtained through selection, the estimates of genetic parameters such as GCV, h^2^, and GA must be taken into consideration. The primary prerequisite for any crop improvement program is the availability of genetic variability. By creating random mutations within the target crop genome, mutation breeding is a coherent tool for improving genetic variability. The estimate of GCV and h^2^ of polygenic traits is very important in predicting the stability of the trait under the influence of environmental factors and possible expressiveness in the next generations [75,76]. The present study shows that mutagenic treatments of gamma rays and sodium azide used individually or in combination caused a considerable increase in GCV, h^2^, and GA in the treated population. The magnitude of GCV was found to be different in different treatments. GCV, h^2^ and GA, may provide the best image of the degree of improvement predicted through phenotypic selection [35,50]. The results showed a wide range of inter-trait variability and a manifold increase in GCV, h^2^, and GA in all the mutagen treatments. The results were in propinquity with the earlier studies in faba bean [77], lentil [50], black cumin [29], cowpea [35], and urd bean [78] that reported increased GCV, h^2^, and GA in mutagenized populations. The analysis of genetic parameters confirmed the possibility of considerable improvement in yield and yield-attributing traits. Thus, increased heritability unveiled the least influence of environmental factors on the expressivity of such polygenic traits, leading to maximum selection gain. Higher h^2^, GCV and GA for traits such as fertile branches, capsules, seeds, seed weight, and seed yield revealed that polygenic additive genes control these quantitative traits, and appropriate selection of all these traits would yield maximum genetic gain [79]. Thus, maximum h^2^ and GA, along with low GCV for traits such as days to flowering, plant height, and days to maturity, showed that all these traits are governed by non-additive genes [79], and selection of these traits would not be beneficial. Maximum h^2^ correlated with GA will promote the productive phenotypic selection of desirable mutant lines [35]. A broad variability induced in yield and yield-attributing traits in a mutagenized population of two linseed varieties in M_2_ generation was recorded, leading to opportunities for a successful selection of desirable mutants. Many workers use induced mutagenesis for the successful selection of desired mutant lines in various crops such as linseed [80], black gram [81], and cowpea [35].

In the present study, high h^2^ obtained for seed yield and yield-attributing traits indicated that selection was effective in the earlier generations and might result in isolating high-yielding mutants in advanced generations. The results were in good agreement with the earlier findings in cowpeas, which reported high h^2^ for seed yield and yield-attributing traits [35]. The evaluation of GA represents the magnitude of the genetic gain obtained for a specific trait after continuous selection through both generations. According to previous studies, h^2^ and GA are reliable indicators for trait selection [82]. Therefore, traits with high h^2^ and GA are ideal for effective selection and subsequent crop improvement. In contrast to the GCV in the successive generation of mutagen-treated population, h^2^ and GA are evaluated, which gives more insights into crop mutations [83].

### 4.4 Correlation analysis: insights into trait interrelationships

In breeding programs, it is critical to have knowledge and broader understanding of the relationship of traits with the total yield. This allows the breeders to prioritise different sets of traits in the direct and indirect selection of high performing mutants. Correlation analysis also provides an idea about role of different traits in determining the overall yield and it is always recommended to prioritise the trait as per their correlation coefficient. Higher correlation coefficient indicates greater contribution of trait toward yield. Therefore, a trait that shows a significant coefficient with the yield, it is better to choose indirect selection, as improving this trait could enhance the yield [84]. Previous studies have also shown the importance of correlation analysis in crop improvement programs [85]. In the present study, a maximum correlation between the harvest index and the number of fertile branches and seeds per capsule reveals that these traits must be prioritized in indirect selection programs.

### 4.5 Conclusion and future directions

The present study reveals that lower doses of mutagens effectively increased the mean values of important traits in linseed. Therefore, these doses could also prove beneficial in future linseed breeding programs. A considerable increase in the GCV, h^2^, and GA induced in the lower and moderate mutagen dose-treated plants also reflected the role of different mutagen doses in altering the genetics of linseed. Most importantly, a few desired putative mutants were isolated in M_2_ generation that could serve as a valuable genetic resource for improving existing linseed varieties and used as parents in the crossbreeding programs. However, we are trying to grow such putative mutants in multi-location trials, to assess their performance and the possibility of registration and release as a new high-yielding linseed variety.

## Data availability statement

Data will be made available on request.

## CRediT authorship contribution statement

**Roshan Jahan:** Writing – original draft, Methodology, Formal analysis. **Aamir Raina:** Writing – review & editing, Writing – original draft, Visualization, Validation, Software, Resources, Formal analysis, Data curation. **Saima Malik:** Software, Resources, Formal analysis, Data curation. **Samiullah Khan:** Supervision, Methodology, Investigation, Data curation, Conceptualization.

## Declaration of competing interest

The authors declare that they have no known competing financial interests or personal relationships that could have appeared to influence the work reported in this paper.
