# Supplementary material for: Gamma rays and sodium azide induced variations in bio-physiological and agronomical traits in linseed (Linum usitatissimum L.)
Source: Heliyon. 2024 May 16;10(11):e31329. doi: 10.1016/j.heliyon.2024.e31329 (PMC11153108; doi:10.1016/j.heliyon.2024.e31329)
Supplement: Multimedia component 1 [file mmc1.docx]

**Supplementary Table 1:** Description of selected linseed varieties.

| **S. No.** | **Variety** | **Place of Release** | **Distinguishing Characters** |
| --- | --- | --- | --- |
| 1. | Var. Padmini | Bundelkhand | Maturity 140-148 days; 1000 seed weight 7.25g; large seed size |
| 2. | Var. IC0096650 | Maharashtra | Maturity 155-160 days; 1000 seed weight 5-6g; small seed size |

**Supplementary Table 2:** Description of mutant lines grown/selected in generation from M_1_ in the var. Padmini

| **Code** | **Mutagen** | **M_1_** | | |
| --- | --- | --- | --- | --- |
|  |  | **Seed Treated** | **Seeds Germinated** | **Fertile Plants** |
| C | **Control*** | 0 | 294 | 279 |
| G1 | **50 Gy γ rays** | 300 | 284 | 256 |
| G2 | **100 Gy γ rays** | 300 | 277 | 233 |
| G3 | **150 Gy γ rays** | 300 | 258 | 226 |
| G4 | **200 Gy γ rays** | 300 | 241 | 192 |
| S1 | **0.1% SA** | 300 | 276 | 252 |
| S2 | **0.2% SA** | 300 | 271 | 223 |
| S3 | **0.3% SA** | 300 | 261 | 235 |
| S4 | **0.4% SA** | 300 | 247 | 188 |
| G1+S1 | **50 Gy γ rays+0.1% SA** | 300 | 270 | 231 |
| G2+S2 | **100 Gy γ rays+0.2% SA** | 300 | 257 | 209 |
| G3+S3 | **150 Gy γ rays+0.3% SA** | 300 | 226 | 166 |
| G4+S4 | **200 Gy γ rays+0.4% SA** | 300 | 219 | 164 |
| T | **Total** | **3600** | **3381** | **2854** |

*For control set, 300 seeds were sown in the same field. ns: no selection.

**Supplementary Table 3:** Description of mutant lines grown/selected in generation from M_1_ in the var. IC0096650.

| **Code** | **Mutagen** | **M_1_** | | |
| --- | --- | --- | --- | --- |
|  |  | **Seed Treated** | **Seeds Germinated** | **Fertile Plants** |
| C | **Control*** | 0 | 289 | 265 |
| G1 | **50 Gy γ rays** | 300 | 279 | 243 |
| G2 | **100 Gy γ rays** | 300 | 274 | 216 |
| G3 | **150 Gy γ rays** | 300 | 252 | 210 |
| G4 | **200 Gy γ rays** | 300 | 239 | 187 |
| S1 | **0.1% SA** | 300 | 271 | 234 |
| S2 | **0.2% SA** | 300 | 266 | 208 |
| S3 | **0.3% SA** | 300 | 259 | 207 |
| S4 | **0.4% SA** | 300 | 236 | 180 |
| G1+S1 | **50 Gy γ rays+0.1% SA** | 300 | 259 | 214 |
| G2+S2 | **100 Gy γ rays+0.2% SA** | 300 | 249 | 199 |
| G3+S3 | **150 Gy γ rays+0.3% SA** | 300 | 224 | 152 |
| G4+S4 | **200 Gy γ rays+0.4% SA** | 300 | 210 | 151 |
| T | **Total** | **3600** | **3307** | **2666** |

* For control set, 300 seeds were sown in the same field. ns: no selection

**Supplementary Table 4:** List of phenotypic traits and description of their quantitative traits measurements.

| **S. No.** | **Traits** | **Description** |
| --- | --- | --- |
| 1. | Plant height (cm) | Plant height measured from the base up to the apex of the plant. |
| 2. | Days to flowering | Days taken from the sowing to flower opening |
| 3. | Days to maturity | Days taken from sowing till the date of harvesting of the plant |
| 4. | Number of fertile branches | Number of fertile branches per plant at maturity |
| 5. | Capsules per plant | Number of capsules per plant at maturity |
| 6. | Seeds per capsule | Number of seeds in a capsule |
| 7. | 1000-seed weight (g) | Weight of a random sample of 1000 seeds from each plant |
| 8. | Seed yield per plant (g) | Weight of total number of seeds harvested per plant |
| 9. | Harvest index (%) | The ratio of seed yield to *biological yield |

*Biological yield (biomass) –Total dry weight of the harvested plant (g per plant).

**Supplementary Figure 1:** Layout of field plots in M_1_ generation with a Complete Randomized Block Design for both the varieties.


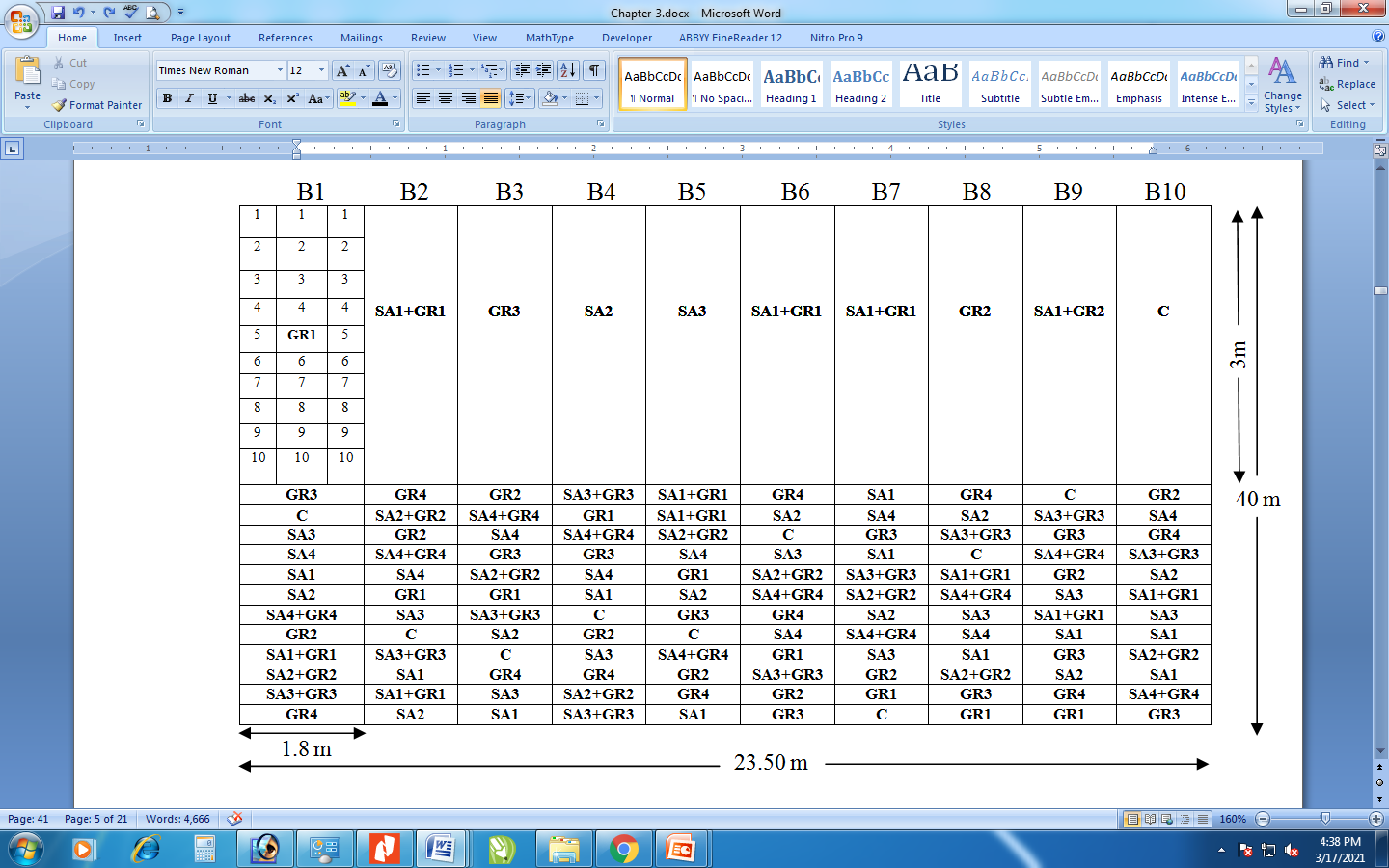


**#**B1= Block1, B2= Block2, B3= Block3, B4= Block4, B5= Block5, B6= Block6, B7= Block7, B8= Block8, B9= Block9, B10= Block10; C= Control, SA1= 0.1% SA, SA2= 0.2% SA, SA3= 0.3% SA, SA4= 0.4% SA, GR1= 50Gy, GR2= 100Gy, GR3= 150Gy, GR4= 200Gy, SA1+GR1= 0.1% SA+50Gy, SA2+GR2= 0.2% SA+100Gy, SA3+GR3= 0.3% SA+150Gy, SA4+GR4= 0.4% SA+200Gy.

**
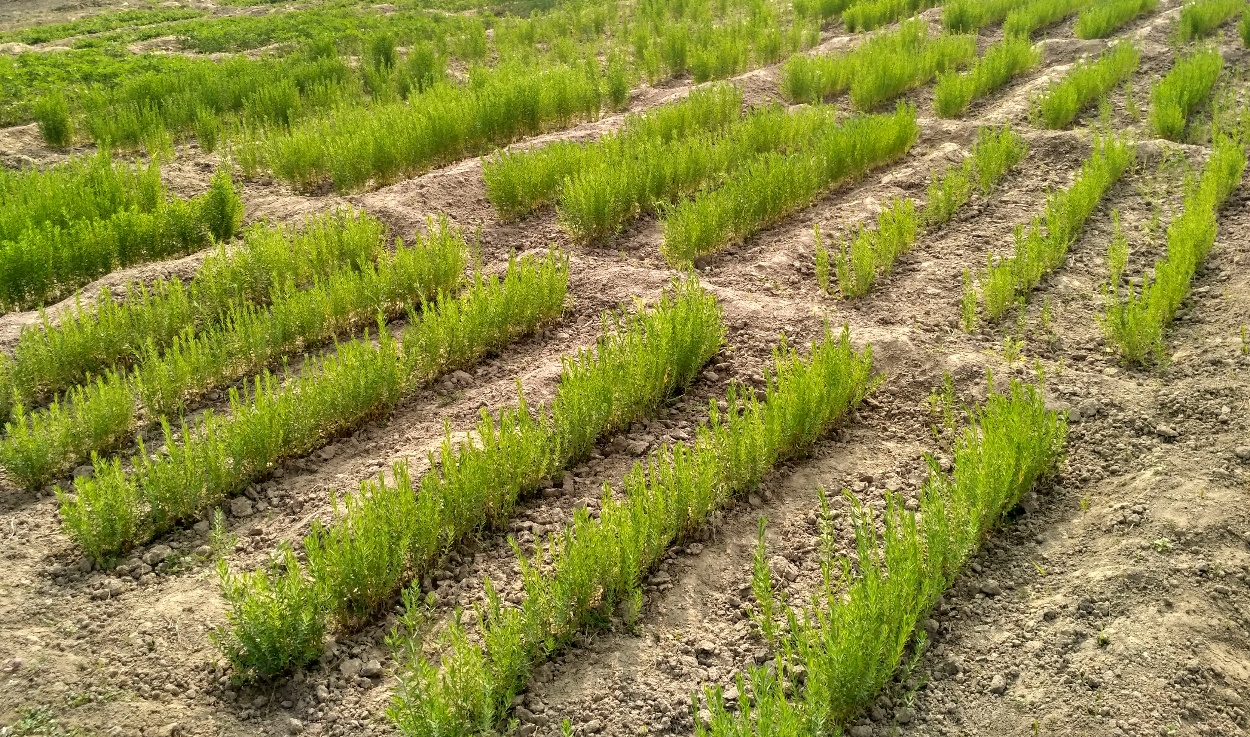
**

Supplementary Figure 2. A picture of the experimental field showing linseed crop at intermediate growth period.
